# Feeder-free differentiation of cells exhibiting characteristics of corneal endothelium from human induced pluripotent stem cells

**DOI:** 10.1242/bio.032102

**Published:** 2018-04-23

**Authors:** Michael D. Wagoner, Laura R. Bohrer, Benjamin T. Aldrich, Mark A. Greiner, Robert F. Mullins, Kristan S. Worthington, Budd A. Tucker, Luke A. Wiley

**Affiliations:** 1 Cornea Research Unit, Department of Ophthalmology & Visual Sciences, Carver College of Medicine, University of Iowa, Iowa City, IA 52242, USA; 2 Institute for Vision Research, Department of Ophthalmology & Visual Sciences, Carver College of Medicine, University of Iowa, Iowa City, IA 52242, USA; 3 Department of Ophthalmology & Visual Sciences, Carver College of Medicine, University of Iowa, Iowa City, IA 52242, USA; 4 Iowa Lions Eye Bank, Coralville, IA 52241, USA; 5 Department of Biomedical Engineering, University of Iowa, Iowa City, IA 52242, USA

**Keywords:** Induced pluripotent stem cells, Differentiation, Neural crest cells, Corneal endothelial cells

## Abstract

The purpose of this study was to devise a strategy for the derivation of corneal endothelial cells (CEnCs) from adult fibroblast-derived induced pluripotent stem cells (iPSCs). IPSCs were generated from an adult human with normal ocular history via expression of *OCT4*, *SOX2*, *KLF4* and *c-MYC*. Neural crest cells (NCCs) were differentiated from iPSCs via addition of CHIR99021 and SB4315542. NCCs were driven toward a CEnC fate via addition of B27, PDGF-BB and DKK-2 to CEnC media. Differentiation of NCCs and CEnCs was evaluated via rt-PCR, morphological and immunocytochemical analysis. At 17 days post-NCC induction, there were notable changes in cell morphology and upregulation of the neural crest lineage transcripts *PAX3*, *SOX9*, *TFAP2A*, *SOX10* and *p75NTR* and the proteins p75/NGFR and SOX10. Exposure of NCCs to B27, PDGF-BB and DKK-2 induced a shift in morphology from a spindle-shaped neural phenotype to a tightly-packed hexagonal appearance and increased expression of the transcripts *ATP1A1*, *COL8A1*, *COL8A2*, *AQP1* and *CDH2* and the proteins ZO-1, N-Cad, AQP-1 and Na^+^/K^+^ATPase. Replacement of NCC media with CEnC media on day 3, 5 or 8 reduced the differentiation time needed to yield CEnCs. IPSC-derived CEnCs could be used for evaluation of cornea endothelial disease pathophysiology and for testing of novel therapeutics.

## Abstract (precis)

**Summary:** This study demonstrates differentiation of adult dermal fibroblast-derived iPSCs into neural crest and corneal endothelial-like cells, which could be a valuable tool for investigating the mechanisms of cornea endothelial disease.

## INTRODUCTION

Disorders of the corneal endothelium are a common cause of ocular morbidity and vision loss. This endothelial dysfunction may occur as a result of hereditary conditions, such as Fuchs' endothelial dystrophy (Stamler et al., 2013) or from degeneration related to aging, trauma, environmental and pharmacologic toxins, infection, or inflammation. Irrespective of cause, the final common pathway of all of these disorders is endothelial attrition which is associated with progressive stromal and epithelial edema and loss of corneal clarity (Smolin et al., 2005).

Endothelial attrition-related vision loss is the leading indication for corneal transplantation in the USA accounting for at least 56% of all such procedures (America EBAO. Eye Bank Association of America, 2016; Park et al., 2015). Transplants performed for this indication are associated with a high probability of success (Anshu et al., 2012b; Smolin et al., 2005), with more than 90% of grafts remaining clear for at least 5 years (Price et al., 1991; Price et al., 2011; Thompson et al., 2003).

The delayed and difficult visual rehabilitation after penetrating keratoplasty (i.e. full-thickness resection of the cornea using a trephine) (Smolin et al., 2005) has been largely circumvented by the relative quick and simple visual improvement that can be offered with endothelial keratoplasty procedures (Koenig et al., 2007; Smolin et al., 2005), particularly Descemet membrane endothelial keratoplasty which achieves anatomic restoration of native corneal structure. Nonetheless, the emergence of endothelial keratoplasty as the treatment of choice for these disorders has not eliminated the need for utilization of cadaveric corneal donation. The use of topical corticosteroids is required to reduce the risk of immunologic rejection and graft failure (Anshu et al., 2012a; Price et al., 2016; Sugar et al., 2009), but their use is associated with an increased risk of developing glaucoma or infectious complications (Al-Mohaimeed et al., 2007; Greenlee and Kwon, 2008; Maier et al., 2013; Mannis and Holland, 2016; Wandling et al., 2012; Ward et al., 2014).

The remaining challenges toward provision of near-perfect visual rehabilitation for patients with corneal endothelial disorders can be addressed by self-provision of endothelial cells from a non-ocular source, such as skin-derived patient-specific induced pluripotent stem cells (iPSCs), for use in endothelial transplantation. The ability to generate virtually unlimited supplies of corneal endothelial cells (CEnCs) from patients is a promising avenue for patient-specific corneal transplantations and would circumvent the need to use post-surgical immunosuppressants. Moreover, the ability to make cells from patients with hereditary disorders could also help improve our understanding of the pathophysiologic mechanisms of cell loss, and in turn be used to evaluate the efficacy of novel gene and drug based therapeutics for development of non-surgical treatment-based strategies.

With the recent explosion in the use of human pluripotent stem cells, we and many others have demonstrated that skin-derived iPSCs can be differentiated into numerous eye-specific cell types, including retinal photoreceptors (Sharma et al., 2017; Wiley et al., 2016b; Zhong et al., 2014), ganglion cells (Sluch et al., 2015; Tucker et al., 2014), retinal-pigmented epithelial cells (Singh et al., 2013; Tucker et al., 2015) and choroidal vascular endothelial cells (Songstad et al., 2015, 2017). In this study we demonstrate the development of CEnCs from an adult control human iPSC line using a novel step-wise differentiation paradigm.

## RESULTS

### Dual inhibition of GSK-3 and TGFβ/SMAD signaling promotes differentiation of iPSCs to neural crest cells

Much of the anterior segment of the eye, including the interior layers of the cornea (i.e. the stroma and endothelium), develop from neural crest-derived mesenchymal cells that migrate into the developing mammalian eye (Cvekl and Tamm, 2004; Seo et al., 2017; Sowden, 2007). Thus, our first goal was to differentiate a human iPSC line into neural crest cells (NCCs). To do this we took advantage of a previously published protocol by Menendez et al. that describes lineage-specific differentiation of human pluripotent stem cells to multipotent neural crest stem cells using two small-molecule compounds: CHIR99021, a GSK-3 inhibitor and SB431542, a TGFβ/SMAD pathway inhibitor (Menendez et al., 2013).

**Fig. 1.**
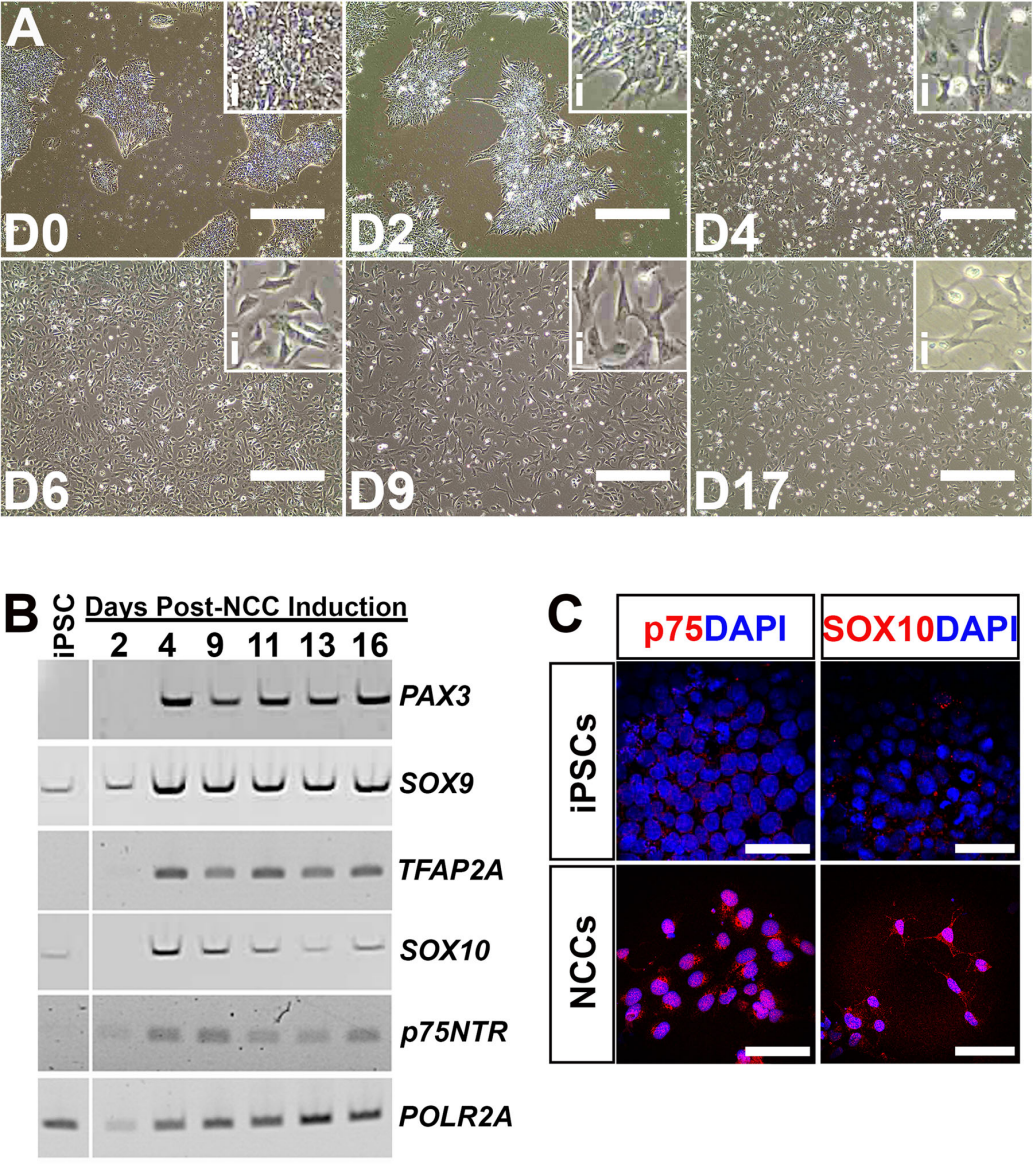
**Differentiation of induced pluripotent stem cells into neural crest cells.** (A) Representative light micrographs of induced pluripotent stem cells differentiating into neural crest cells at 0 (D0), 2 (D2), 4 (D4), 6 (D6), 9 (D9) and 17 (D17) days post-NCC induction. Insets (i) in each panel show higher magnification views to better display changes in cell morphology throughout the differentiation process. (B) rt-PCR for NCC-specific transcripts comparing undifferentiated iPSCs to cells at 2, 4, 9, 11, 13 and 16 days post-NCC induction. *POLR2A* served as a control transcript. (C) Immunocytochemical labeling of undifferentiated iPSCs and NCCs at 16 days post-induction with the NCC-specific markers, p75/NGFR (red) and SOX10 (red). DAPI was used to counterstain cell nuclei. Scale bars: 400 µm in A; 40 µm in C.

Culture of human control iPSCs in a medium containing 3 µM CHIR99021 and 10 µM SB431542 over the course of 17 days led to a loss of iPSC colonization and a shift in morphology from cells with a high nucleus-to-cytoplasmic ratio to a more elongated structure with a neuronal morphology (Fig. 1A). Changes in cell morphology were concomitant with expression of transcription factors that are known to promote a neural crest lineage such as *PAX3*, *SOX9*, *TFAP2A*, *SOX10* and *p75NTR* (Fig. 1B). At 17 days post-induction, neural crest cells displayed positive labeling for the markers p75/NGFR and SOX10 compared to undifferentiated iPSCs which were negative for each marker (Fig. 1C). These results demonstrate that we achieved successful directed differentiation of iPSCs into neural crest cells.

### Differentiation of neural crest cells into corneal endothelial cells

**Fig. 2.**
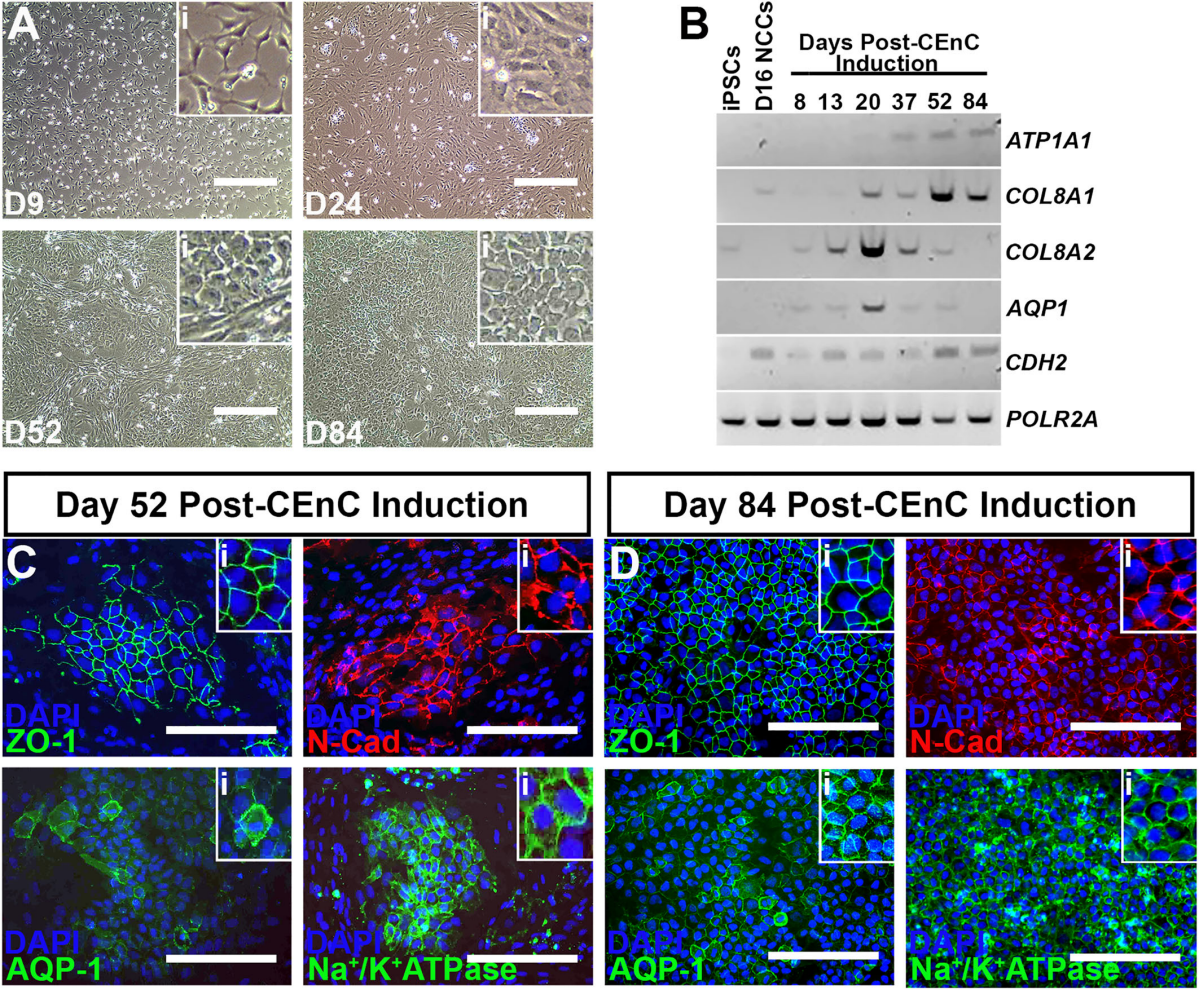
**Differentiation of neural crest cells into corneal endothelial cells.** (A) Representative light micrographs of neural crest cells differentiating into corneal endothelial cells at 9 (D9), 24 (D24), 52 (D52) and 84 (D84) days post-CEnC induction. Insets (i) in each panel show higher magnification views to better display changes in cell morphology throughout the differentiation process. (B) Rt-PCR for CEnC-specific transcripts comparing undifferentiated iPSCs and day 16 NCCs (D16 NCCs) to differentiated cells at 8, 13, 20, 37, 52 and 84 days post-CEnC induction. *POLR2A* served as a control transcript. (C) Representative immunocytochemical labeling of differentiated cells at 52 days post-CEnC induction with the CEnC-specific markers, zonula occludens-1 (upper left; ZO-1, green), N-Cadherin (upper right; N-Cad, red), Aquaporin-1 (lower left; AQP-1, green) and Na^+^/K^+^ATPase (lower right, green). DAPI was used to counterstain cell nuclei. Insets (i) in each panel show higher magnification views. (D) Representative immunocytochemical labeling of differentiated cells at 84 days post-CEnC induction with ZO-1 (upper left, green), N-Cad (upper right, red), AQP-1 (lower left, green) and Na^+^/K^+^ATPase (lower right, green). DAPI was used to counterstain cell nuclei. Insets (i) in each panel show higher magnification views. Scale bars: 400 µm in A; 200 µm in C,D.

To test whether iPSC-derived neural crest cells could be driven towards a corneal endothelial cell fate, we began by culturing day 17 NCCs in a medium augmented with the neural growth supplement, B27, the endothelial mitogen, PDGF-BB and the WNT signaling inhibitor, DKK-2, a formulation previously described by McCabe et al. (McCabe et al., 2015). After 9 days in CEnC-induction medium cells retained a NCC-like morphology, but by day 24 many cells began to adopt a more cobblestone-like appearance, which was consistently observed through day 52 post-differentiation (Fig. 2A). By 84 days post-CEnC differentiation, cells formed tightly-packed sheets of hexagonal cells (Fig. 2A). Assessment of mRNA via rt-PCR showed that cells expressed the CEnC-specific transcripts *ATP1A1*, *COL8A1*, *COL8A2*, *AQP1* and *CDH2* as early as 20 days post-CEnC induction and this expression persisted to the 84-day timepoint (Fig. 2B). Next, we validated the observed upregulation in expression of CEnC-specific transcripts via immunocytochemical evaluation of CEnC-specific protein markers in differentiated cells at 52 and 84 days post-CEnC induction. At day 52 of CEnC differentiation, we observed pockets of tightly-packed polygonal CEnC precursor cells that expressed the tight junction protein, zonula occludens-1 (ZO-1), the adherens junction protein, N-Cadherin (N-Cad), the water channel protein, Aquaporin-1 (AQP-1) and the enzyme pump, Na^+^/K^+^ATPase (Fig. 2C). Expansion of differentiation to 84 days yielded more widespread sheet-like cultures greater than 95% of which were immunopositive for CEnC-specific proteins (Fig. 2D), consistent with cultured human donor CEnCs (He et al., 2016; Parekh et al., 2017). Moreover, day 84 iPSC-CEnCs were negative for cornea epithelial-specific markers (Fig. S1). Together, these data establish successful derivation of corneal endothelial cells from patient-derived human pluripotent stem cells.

### Differentiating corneal endothelial cells can withstand process of freezing and thawing

**Fig. 3.**
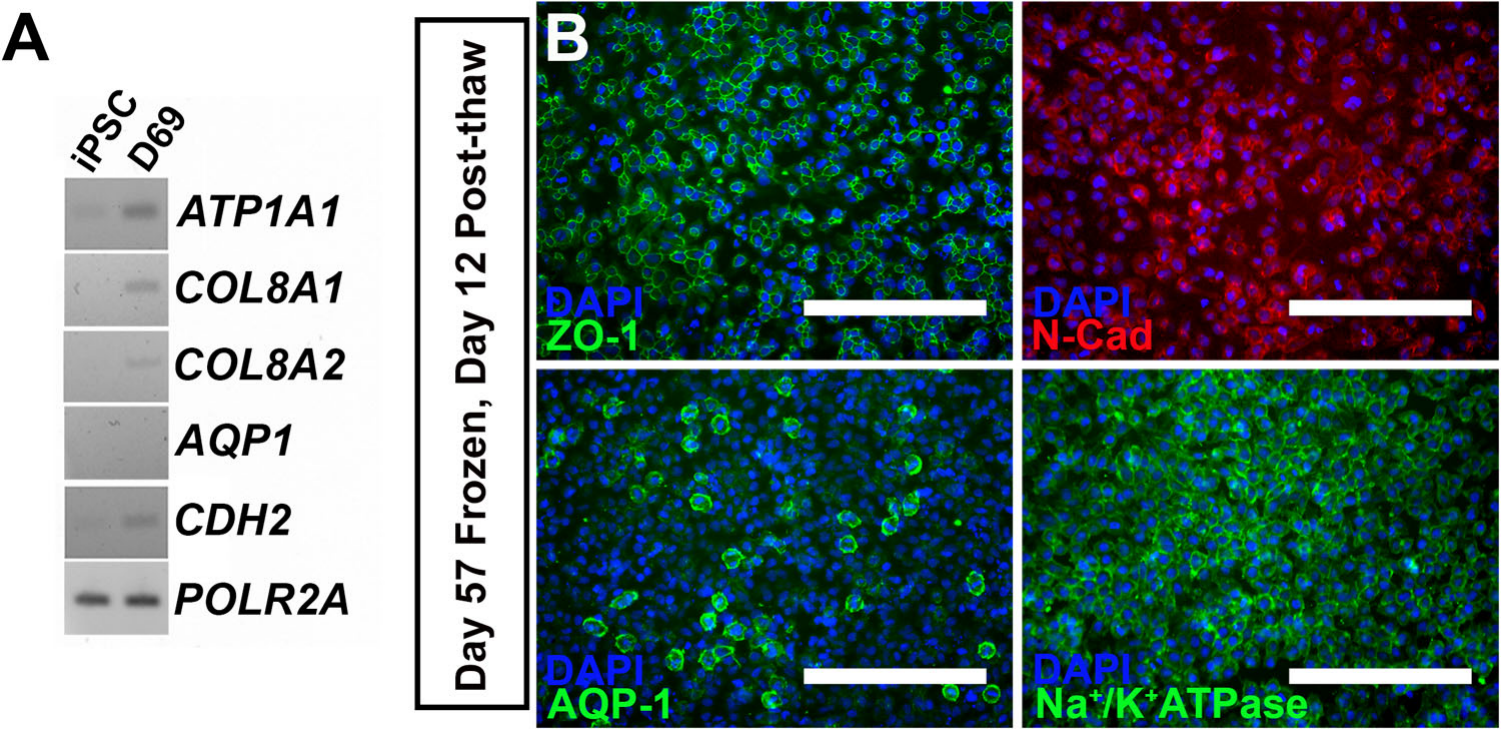
**IPSC-derived corneal endothelial cells retain expression of CEnC markers following cryostorage, thaw and re-culture.** (A) Rt-PCR for CEnC-specific transcripts comparing undifferentiated iPSCs and iPSC-derived CEnCs that were frozen at day 57 post-CEnC induction, thawed and differentiated in CEnC induction medium for 12 days (69 days total post CEnC induction). *POLR2A* served as a control transcript. (B) Representative immunocytochemical labeling of frozen/thawed corneal endothelial cells labeled with zonula occludens-1 (upper left; ZO-1, green), N-Cadherin (upper right; N-Cad, red), Aquaporin-1 (lower left; AQP-1, green) and Na+/K+ATPase (lower right, green). Scale bars: 200 µm.

For iPSC-derived CEnCs to be utilized as a source for cell therapy, the ability to differentiate cells at the site of production, freeze, ship to the point of care, and re-derive CEnC cultures for transplantation would be critical. As such, we tested whether differentiating CEnC cells could: (1) survive being frozen and thawed and (2) if cells retained morphology and expression of corneal endothelial-specific proteins. For this experiment we froze cells at 57 days post-CEnC differentiation. At 3 months post-freeze, cells were thawed and re-plated in CEnC-induction medium for 12 days. Thawed cells grew at a rate similar to those before the freezing process and expressed CEnC-specific transcripts as detected via rt-PCR (Fig. 3A) and displayed tightly-packed CEnC morphology and expression of ZO-1, N-Cad, AQP-1 and Na^+^/K^+^ATPase protein (Fig. 3B). These data suggest that cells can be arrested during the differentiation process without loss of corneal endothelial cell lineage specification.

### The timeline of CEnC induction can be shortened by using early-stage NCCs

**Fig. 4.**
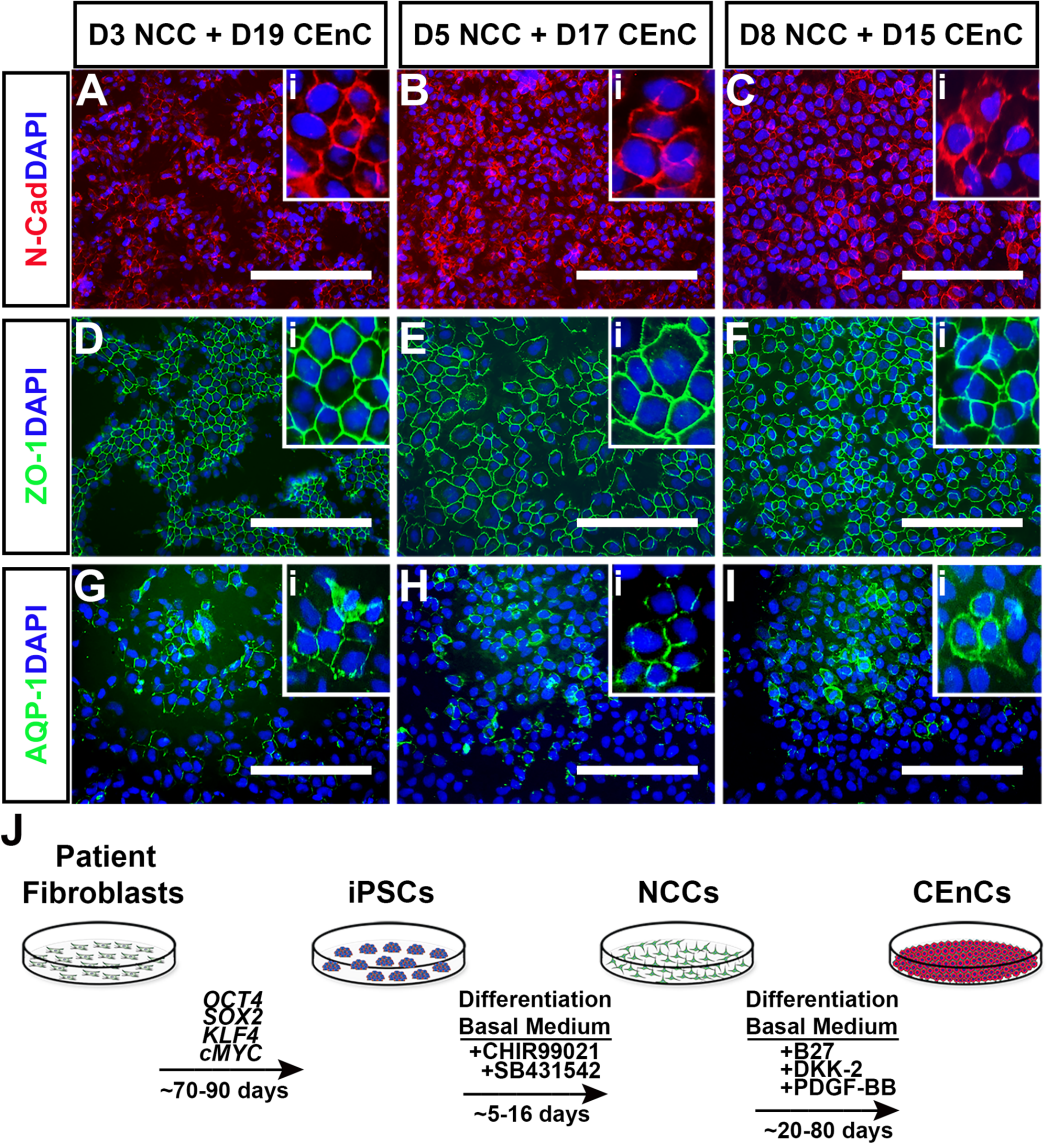
**Derivation of iPSC-derived corneal endothelial cells does not require maturation of neural crest cells.** (A-I) Representative immunocytochemical analysis of cells labeled with N-Cadherin (A-C; N-Cad, red), zonula occludens-1 (D-F; ZO-1, green) and Aquaporin-1 (G-I; AQP-1, green). Cells were assessed for the presence of corneal endothelial markers following initial differentiation for 3 (D3; A,D,G), 5 (D5; B,E,H) and 8 (D8; C,F,I) days in NCC-induction medium followed by subsequent differentiation in CEnC-specific medium for 19 (D19; A,D,G), 17 (D17; B,E,H) and 15 (D15; C,F,I) days, respectively. Insets (i) in each panel show higher magnification views. DAPI was used to counterstain cell nuclei. Scale bars: 200 µm. (J) Schematic summarizing the step-wise differentiation paradigm for the generation of patient-specific corneal endothelial cells. Fibroblasts derived from a patient-specific skin biopsy are reprogrammed into pluripotent stem cells (iPSCs) via exogenous expression of the well-documented transcription factors, *OCT4*, *SOX2*, *KLF4* and *cMYC*. This process, including isolation and expansion of iPSC colonies typically takes ∼70-90 days. The initial step towards derivation of corneal endothelial cells is differentiation of iPSCs to neural crest cells (NCCs) with treatment of cells with the GSK-3 inhibitor, CHIR99021 and the TGFβ/SMAD inhibitor, SB431542 for ∼5-16 days. NCCs were then terminally differentiated into corneal endothelial cells (CEnCs) using the same differentiation basal medium supplemented with B27, PDGF-BB and the WNT signaling inhibitor, DKK-2 for ∼50-80 days.

As shown in Figs 1 and 2, derivation of NCCs and subsequent CEnC specification took over 50 days of differentiation. In an attempt to reduce differentiation time, we asked if we could reduce the NCC induction period prior to CEnC induction. To that end, we differentiated iPSCs in NCC-induction medium for 3, 5 or 8 days before switching them to CEnC-induction medium for an additional 15-20 days (Fig. 4). Corneal endothelial induction following 3 (Fig. 4A,D,G), 5 (Fig. 4B,E,H) or 8 (Fig. 4C,F,I) days of neural crest differentiation produced CEnC cells that expressed N-Cad, ZO-1 and AQP-1. These results show that the timeframe of our iPSC-CEnC differentiation paradigm can be reduced from more than ∼50 days to ∼25 days by restricting neural crest cell induction to 3-8 days (Fig. 4J).

## DISCUSSION

In this study, we demonstrate successful differentiation of neural crest cells and corneal endothelial cells from patient-specific iPSCs derived from dermal fibroblasts isolated from an adult male patient with a normal ocular history. We describe a paradigm that utilizes a small molecule approach to drive directed development of neural crest cells and subsequent generation of corneal endothelial cells in a feeder-free manner, as summarized in Fig. 4J. IPSC-derived corneal endothelial cells display a hexagonal, tightly-packed morphology and express transcripts and proteins that are established markers of mature corneal endothelial cells (De Roo et al., 2017; He et al., 2016; Parekh et al., 2017).

Other groups have also reported the generation of corneal endothelial cells from human iPSCs (Table 1) (McCabe et al., 2015; Zhao and Afshari, 2016). However, each of these protocols, including the one we describe in this report, are distinct with regard to the type of pluripotent cells used for derivation, the culture medium used for maintenance and differentiation of cells, the small molecules and growth factors used for neural crest or corneal endothelial cell induction and the time each protocol takes to generate mature CEnC cells. McCabe et al. described the use of pluripotent embryonic stem cells (ESCs) derived from human blastocysts and dual Smad inhibition via SB431542 and Noggin to induce neural crest cell formation in two days (McCabe et al., 2015). Like our approach, CEnC induction was achieved via administration of human recombinant PDGF-BB and the Wnt-specific pathway inhibitor, DKK-2, resulting in corneal endothelial cells in approximately 10 days. In contrast, we recapitulated the derivation of neural crest cells described by Dalton and colleagues (Menendez et al., 2013). Specifically, instead of Noggin, an inhibitor of the TGFβ pathway, we treated iPSCs with the GSK-3-specific inhibitor, CHIR99021, which acts to block the influences of signaling from mitogen-activated protein kinases to prevent terminal differentiation of cells (Sato et al., 2004). As shown in the results above, administration of SB431542 and CHIR99021 produced neural crest cells in as little 3 days and a neural crest cell phenotype could be maintained for roughly 17 days. Our initial differentiation attempts took more than 50 days in the presence of PDGF-BB and DKK-2 to achieve CEnC cells (Fig. 2), suggesting that prolonged exposure to NCC-inducing factors could be inhibitory to CEnC-induction. In fact, we also demonstrated that using early stage NCCs resulted generation of CEnC cells in only ∼25 days compared to the use of later stage NCCs (Fig. 3).

**Table 1.**
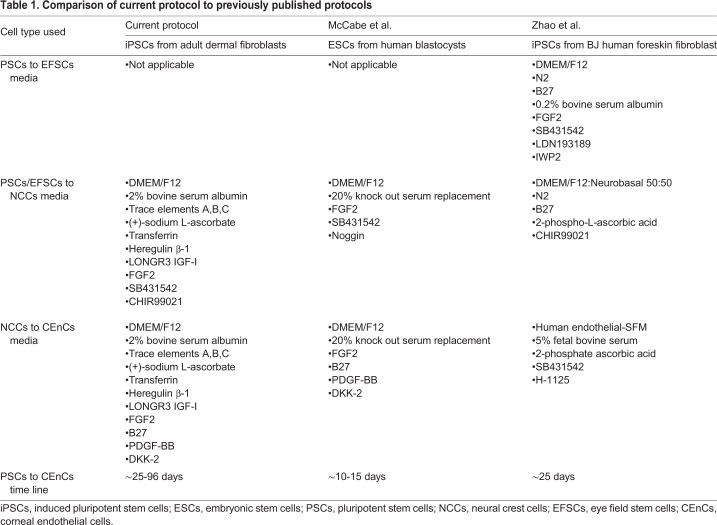
**Comparison of current protocol to previously published protocols**

Recently, Zhao et al. also described the generation of CEnC cells from human iPSCs (Zhao and Afshari, 2016). Interestingly, this approach described the initial differentiation of iPSCs into ‘eye field stem cells’ that expressed the eye lineage-specific transcription factors, PAX6, LHX2 and VSX2, prior to induction of ‘ocular neural crest stem cells’ and subsequent derivation of corneal endothelial cells. This approach raises an important developmental question as to whether the mammalian corneal endothelium is derived from ‘ocular neural crest cells’. However, this concept is in direct contrast to the accepted developmental paradigm that the corneal stroma and endothelium layers are derived from infiltrating cranial mesenchymal neural crest cells (Cvekl and Tamm, 2004; Heavner and Pevny, 2012). However, it is plausible that there is a subset of neural crest cells that are eye specific and transcriptionally-fated to become corneal endothelial cells. Engaging in future experiments to compare the physiological attributes of pan neural crest- versus ocular-specific neural crest-derived CEnCs would be a worthwhile endeavor.

Furthermore, Zhao et al. described the generation of CEnC cells from iPSCs derived from BJ human foreskin fibroblasts (Zhao and Afshari, 2016). An important aspect of our study is the use of iPSCs derived from an adult patient's dermal fibroblasts. Moreover, we have previously reported that isolation, expansion and culture of dermal fibroblasts, and in turn the generation of iPSCs, is more difficult from aged individuals compared to persons younger in age (Wiley et al., 2016b). This is particularly true when using reagents designed for xeno-free and cGMP-compliant culture of cells (Wiley et al., 2016b), an inevitable requirement for translational of CEnC therapies. The demonstration of successful derivation of corneal endothelial cells from adult fibroblast-derived iPSCs is therefore of significance for older patients with severe corneal endothelial-related disease.

Despite differences between ours and the previously published protocols discussed above, the technology now exists to successfully derive corneal endothelial cells from human pluripotent stem cells. It is conceivable that some combination of each of these approaches would yield the most efficient and reproducible protocol for the generation of CEnCs. Perhaps the biggest remaining unanswered question is: how physiologically similar are stem cell-derived corneal endothelial cells to mature human CEnCs? Future studies should be directed at testing the functionality of stem cell-derived CEnCs by their ability to pump water and metabolites (Hara et al., 2014; Kageyama et al., 2017) and survive the energy demands of these ATP-dependent pump activities (Aldrich et al., 2017; Greiner et al., 2015).

Corneal endothelial disorders are the leading cause of corneal transplants each year in the USA (America EBAO. Eye Bank Association of America, 2016; Park et al., 2015). Although corneal transplants are highly effective in treating ocular morbidity and visual loss related to endothelial failure-related corneal edema (Lee et al., 2009), there are some limitations with the current use of human cadaver-derived allogenic endothelial allografts relative to those that would exist with autograft transplantation. The procurement and distribution of human donor eyes from deceased organ donors through eye bank networks is feasible but tissue quality varies from donor to donor, which may impact outcomes. The use of allogenic tissue requires the use of immunosuppressive therapy, primarily with topical corticosteroids, to help prevent graft rejection episodes or to treat them when they occur (Anshu et al., 2012a; Price et al., 2016; Sugar et al., 2009; Wagoner et al., 2009; 2007b). The use of these medications can be associated with the development of new-onset or worsening of pre-existing glaucoma (Al-Mohaimeed et al., 2007; Greenlee and Kwon, 2008; Maier et al., 2013; Mannis and Holland, 2016; Wandling et al., 2012; Ward et al., 2014) and an increased risk of postoperative graft infections (Wagoner et al., 2009; 2007a). Immunosuppressive therapy is not universally successful in reversing rejection episodes and preventing graft failure (Wagoner et al., 2007a). Many patients require one or more repeat transplant procedures in their lifetime (America EBAO. Eye Bank Association of America, 2016), each of which is associated with a progressively higher risk of failure (Al-Mezaine et al., 2006).

There is a critical need to investigate and find alternative avenues for autologous transplantation for corneal endothelial disorders. Human iPSCs are an obvious potential resource for cell-based therapies with distinct advantages and disadvantages for each well-studied stem cell type (i.e. embryonic or induced pluripotent stem cells). Embryonic stem cells (ESCs) could provide universal donor pools from which to derive relatively unlimited supplies of corneal endothelial cells for transplantation. This would relieve the burden on eye bank networks for cadaveric tissue but would not eliminate the clinical issues of immunological rejection and complications associated with preventative strategies. The use of patient-specific, autologous iPSCs would relieve the immunologic clinical issues, but would be associated with higher costs and require laborious production on a per patient basis. The generation and successful transfer of a sufficient numbers of iPSC-derived endothelial cells to eyes with acquired cell loss, such as those with surgical trauma or previous graft failure, could be expected to provide long-term corneal clarity and visual function while drastically reducing the need for extensive postoperative monitoring and topical therapy relative to current or proposed allogenic procedures. The prognosis would be somewhat less optimistic when the patient-specific iPSCs are generated from an individual with an inherited form of corneal endothelial dystrophy, such as Fuchs endothelial dystrophy (Stamler et al., 2013). In these cases, accelerated endothelial attrition associated with the disease-causing mutations in the cells used for transplantation would impose some limitations on the long-term viability of the transplanted cells and would eventually lead to graft failure and the need to repeat the original intervention. However, genome editing technologies, specifically CRISPR-Cas9-mediated repair, could afford researchers the opportunity to genomically-correct disease-causing mutations in patient-specific iPSCs and thus derive cells for transplantation that are disease free (Burnight et al., 2017), thereby providing an equivalent prognosis to that of acquired disorders.

Another advantage offered by the use of patient-specific iPSCs to generate corneal endothelial cells is the potential to model disease and thereby investigate the pathophysiological mechanism(s) that cause endothelial dystrophies such as Fuchs' endothelial dystrophy. Multiple genetic loci have been linked to Fuchs' endothelial dystrophy, including but not limited to *TCF4*, *COL8A2*, *SLC4A11*, *ZEB1* and *LOXHD1* (Hamill et al., 2013). Although significant effort has been dedicated to identifying the genetic and molecular mechanisms that influence endothelial cell attrition (Azizi et al., 2011; Du et al., 2015; Jurkunas et al., 2010; Matthaei et al., 2015), most studies have been limited by the significant genetic and phenotypic variability that occurs in the patient population. Combining patient-specific iPSC and CRIPSR-Cas9 genome editing technologies, investigators can now gain further insight into the pathophysiology of inherited endothelial dystrophies through rigorous disease modelling studies using isogenic patient cells (i.e. CRISPR-corrected versus non-CRISPR-corrected lines that will be genomically-matched cells that will differ only by the presence of a disease-causing mutation). This approach would be a powerful means to study cell physiological aberrations implicated in corneal endothelial disease such as mitochondrial dysfunction and oxidative stress (Gendron et al., 2016; Greiner et al., 2015; Guha et al., 2017; Kim et al., 2017), epithelial-to-mesenchymal transition (Du et al., 2015; Matthaei et al., 2015; Okumura et al., 2015), or cellular signaling pathways like transforming growth factor-beta (De Roo et al., 2016) or the unfolded protein response (Okumura et al., 2017). Carefully designed and rigorous studies using CRISPR-Cas9-corrected, isogenic patient-specific iPSC-CEnCs could lead to new breakthroughs in the understanding of cornea endothelial dystrophies and could give rise to new avenues of therapy.

## MATERIALS AND METHODS

### Patients and ethics statement

All patients provided written, informed consent for this study, which was approved by the Institutional Review Board of the University of Iowa (project approval #199904167) and adhered to the tenets set forth in the Declaration of Helsinki.

### Generation of patient-specific iPSCs

A 3 mm skin biopsy was obtained from the lower leg of a 45-year-old individual with normal ocular history and used for generation of patient-specific induced pluripotent stem cells (iPSCs) as described previously (Sharma et al., 2017; Wiley et al., 2016b, 2017). Briefly, 250,000 patient-specific dermal fibroblasts were plated in one well of a six-well culture dish and transduced with non-integrating Sendai viral vectors driving expression of *OCT4*, *SOX2*, *KLF4* and *c-MYC* at a multiplicity of infection of 3 (CytoTune-iPS Reprogramming Kit; Thermo Fisher Scientific). IPSC colonies were cultured under feeder-free conditions on recombinant human Laminin-521-coated plates (Thermo Fisher Scientific) and maintained in Human Essential 8™ media (Thermo Fisher Scientific) (Wiley et al., 2017). All patient-specific iPSCs are authentic and routinely tested for mycoplasma contamination using the MycoAlert PLUS Mycoplasma Detection Kit (Lonza Walkersville, Inc.,Walkersville, USA).

### Differentiation of patient-specific iPSCs into neural crest and corneal endothelial cells

Adult human iPSCs were passaged using Accutase (Sigma-Aldrich) onto recombinant human Laminin-521-coated plates and the next day the media was switched to differentiation basal medium: DMEM/F12 (Thermo Fisher Scientific), 2% bovine serum albumin (Probumin; Millipore), 2 mM GlutaMAX (Thermo Fisher Scientific), 0.1 mM MEM non-essential amino acid solution (Thermo Fisher Scientific), 1× trace elements A, B, and C (Thermo Fisher Scientific), 0.1 mM 2-mercaptoethanol (VWR; Radnor, USA), 50 µg/ml (+)-sodium L-ascorbate (Sigma-Aldrich), 10 µg/ml transferrin, 10 ng/ml recombinant human Heregulin β-1 (Peprotech; Rocky Hill, USA), 200 ng/ml recombinant human LONGR3 IGF-I (Sigma-Aldrich), 8 ng/ml recombinant human FGF2 (Waisman Biomanufacturing; Madison, USA), 0.2% primocin (InvivoGen; San Diego, USA), plus neural crest cell induction factors [10 µM SB431542 (Cayman Chemical; Ann Arbor, USA) and 3 µM CHIR99021 (Miltenyi Biotec Inc.; Auburn, USA)], as described by Menendez et al. (Menendez et al., 2013) to yield neural crest induction medium. Neural crest induction medium was replaced daily and cells were passaged with Accutase as needed for 3-17 days. Media was then switched to corneal endothelial cell (CEnC) induction medium: differentiation basal media (described above) plus CEnC factors [0.1× B27 (Thermo Fisher Scientific), 10 ng/ml recombinant human PDGF-BB (Peprotech) and 10 ng/ml recombinant human DKK-2 (Peprotech)]. CEnC medium was replaced daily for the first 30 days and then replaced 3 days a week thereafter.

### Immunocytochemistry and fluorescent microscopy

Cells were passaged onto recombinant human laminin-521-coated four-well chamber slides or 24-well plates and grown to desired confluence. Cells were washed in 1× phosphate buffered saline (Thermo Fisher Scientific) and fixed for 10 min in 4% paraformaldehyde at room temperature. For labeling with anti-Na^+^/K^+^ATPase, cells were fixed for 10 min with ice cold 100% methanol at room temperature. Cells were blocked in immunocytochemical blocking buffer as described previously (Wiley et al., 2016a) and were labeled with the following primary antibodies overnight at 4°C: rabbit anti-p75/NGFR (Cell Signaling Technology; Danvers, USA; Cat. No. 8238; 1:500 dilution), rabbit anti-SOX10 (Cell Signaling Technology; Cat. No. 89356; 1:500 dilution), mouse anti-zonula occludens-1 (Thermo Fisher Scientific; Cat. No. 33-9100; 1:500 dilution), rabbit anti-N-Cadherin (Cell Signaling Technology; Cat. No. 13116; 1:250 dilution), mouse anti-Aquaporin-1 (Abcam; Cat. No. ab9566; 1:250 dilution) or mouse anti-Na^+^/K^+^ATPase (Millipore; Cat. No. 05-369; 1:100 dilution). Sections of human cornea or differentiated CEnCs were labeled with rabbit anti-Cytokeratin 12 (Santa Cruz Biotechnology; Cat. No. SC-25722; 1:500 dilution) or mouse anti-Keratin 3/Keratin 76 (Millipore; Cat. No. CBL218-1; 1:500 dilution). Labeled cells were rinsed the next day in wash buffer [1× phosphate buffered saline (Thermo Fisher Scientific), 0.2% Tween^®^ 20 (Sigma-Aldrich)] and subsequently incubated in goat anti-mouse Alexa Fluor 488 (Thermo Fisher Scientific; Cat. No. A-11001) or goat anti-rabbit Alexa Fluor 647 secondary antibody (Thermo Fisher Scientific; Cat. No. A-21245) for 2 h at room temperature in immunocytochemical blocking buffer followed by additional rinses in wash buffer. All antibodies were tested and validated for specific labeling and showed negligible background staining. Cells were then incubated with 4′,6-Diamidino-2-phenylindole dihydrochloride (DAPI) (Thermo Fisher Scientific; Cat. No. 62248; 1:1,000 dilution) for 30 min at room temperature. Labeled cells grown on culture plates were visualized using an EVOS FL microscope (Thermo Fisher Scientific). Labeled cells grown on chamber slides were visualized using a Leica DM 2500 SPE confocal microscope (Leica Microsystems; Wetzlar, Germany).

### Reverse transcription (rt)-PCR

Total RNA was isolated with the RNeasy mini kit (Qiagen) according to the manufacturer's protocol. cDNA was made with the High Capacity cDNA Reverse Transcription kit (Thermo Fisher Scientific) using 100 ng of RNA template. Standard PCR was performed using BIOLASE DNA polymerase (Bioline; Taunton, USA) and primers (Integrated DNA Technologies; Coralville, USA) hybridizing to human transcripts listed in Table 2.

**Table 2.**
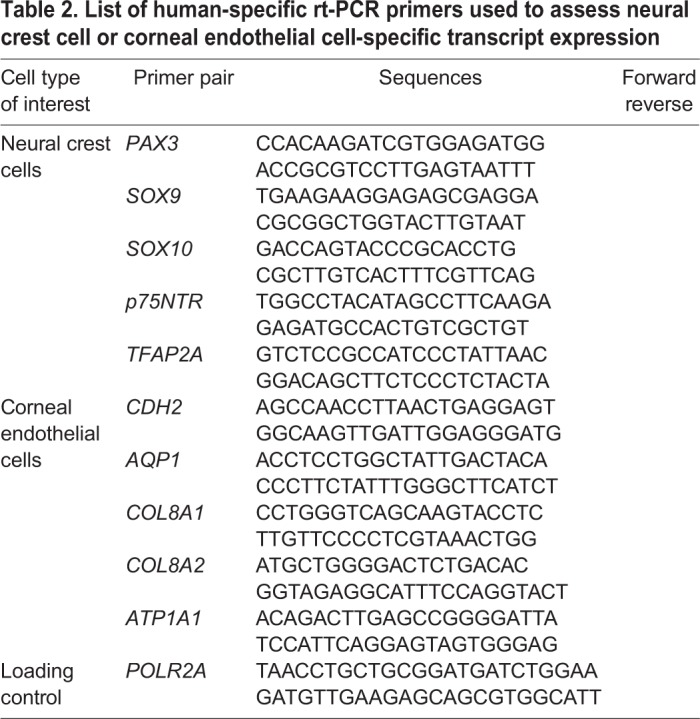
**List of human-specific rt-PCR primers used to assess neural crest cell or corneal endothelial cell-specific transcript expression**

## Supplementary Material

Supplementary information
