# Supplementary material for: Feeder-free differentiation of cells exhibiting characteristics of corneal endothelium from human induced pluripotent stem cells
Source: Biol Open. 2018 Apr 23;7(5):bio032102. doi: 10.1242/bio.032102 (PMC5992532; doi:10.1242/bio.032102)
Supplement: Supplementary information [file biolopen-7-032102-s1.pdf]

Supplemental Data

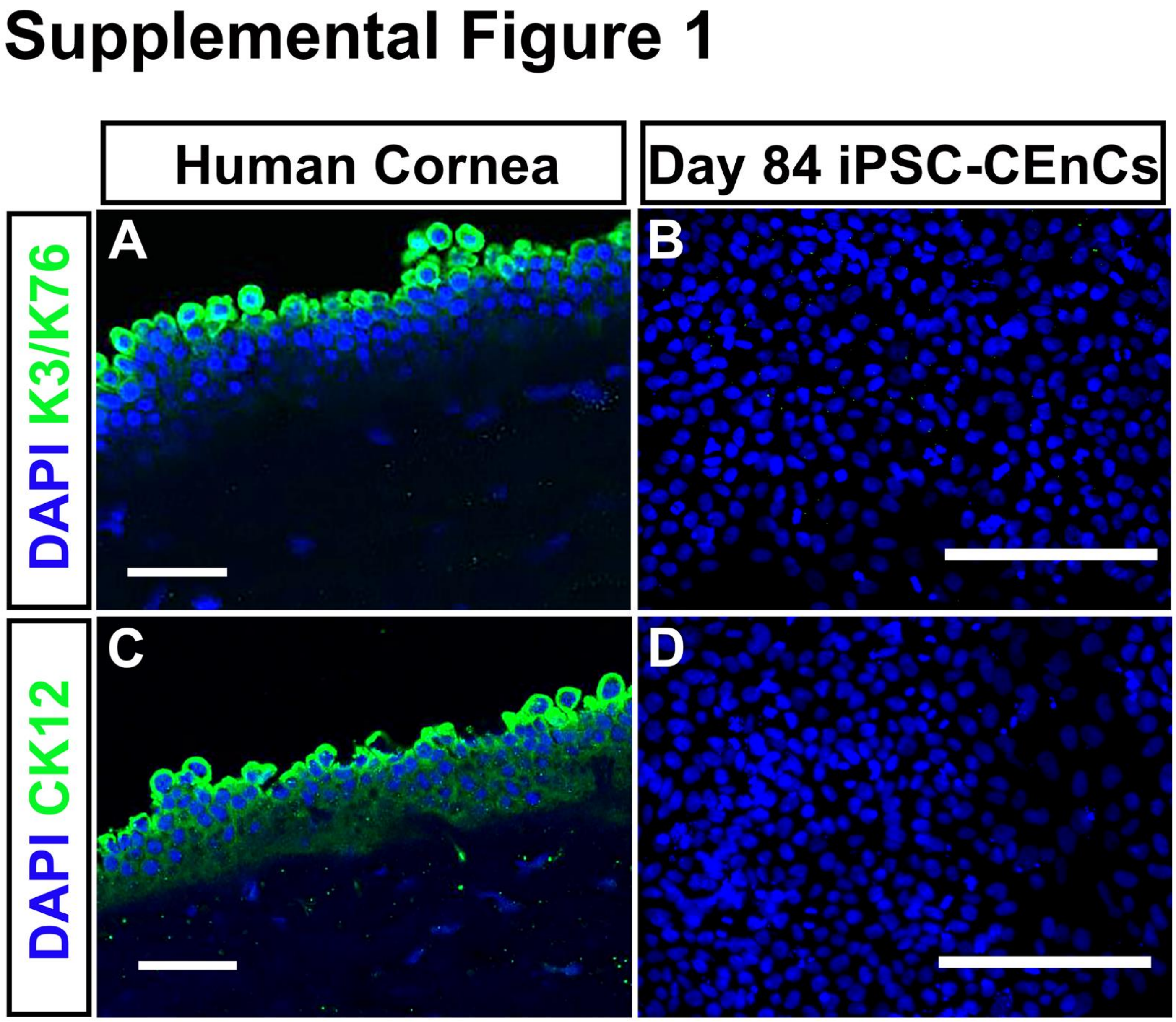

Supplemental Figure 1: iPSC-derived CEnCs do not express markers specific to the corneal epithelium. A-D) Representative immunocytochemical labeling of sections of human cornea (A, C) and Day 84 iPSC-derived CEnCs (B, D) with antibodies against the corneal epithelial-specific markers, keratin 3/keratin 76 (A-B; K3/K76; green) and cytokeratin 12 (C-D; CK12; green). DAPI was used to counterstain cell nuclei. Scale bars = 50 µm in A, C and 200 µm in B, D.
